# Observed increasing water constraint on vegetation growth over the last three decades

**DOI:** 10.1038/s41467-021-24016-9

**Published:** 2021-06-18

**Authors:** Wenzhe Jiao, Lixin Wang, William K. Smith, Qing Chang, Honglang Wang, Paolo D’Odorico

**Affiliations:** 1 Department of Earth Sciences, Indiana University-Purdue University Indianapolis, Indianapolis, IN USA; 2 School of Natural Resources and the Environment, University of Arizona, Tucson, AZ USA; 3 O’Neill School of Public and Environmental Affairs, Indiana University, Bloomington, IN USA; 4 Department of Mathematical Sciences, Indiana University-Purdue University Indianapolis, Indianapolis, IN USA; 5 Department of Environmental Science Policy and Management, University of California, Berkeley, CA USA

**Keywords:** Climate-change ecology, Hydrology

## Abstract

Despite the growing interest in predicting global and regional trends in vegetation productivity in response to a changing climate, changes in water constraint on vegetation productivity (i.e., water limitations on vegetation growth) remain poorly understood. Here we conduct a comprehensive evaluation of changes in water constraint on vegetation growth in the extratropical Northern Hemisphere between 1982 and 2015. We document a significant increase in vegetation water constraint over this period. Remarkably divergent trends were found with vegetation water deficit areas significantly expanding, and water surplus areas significantly shrinking. The increase in water constraints associated with water deficit was also consistent with a decreasing response time to water scarcity, suggesting a stronger susceptibility of vegetation to drought. We also observed shortened water surplus period for water surplus areas, suggesting a shortened exposure to water surplus associated with humid conditions. These observed changes were found to be attributable to trends in temperature, solar radiation, precipitation, and atmospheric CO_2_. Our findings highlight the need for a more explicit consideration of the influence of water constraints on regional and global vegetation under a warming climate.

## Abstract (web summary)

Jiao et al. conducted a comprehensive evaluation of changes in water constraint on vegetation growth in the extratropical Northern Hemisphere between 1982 and 2015. They document a significant increase in vegetation water constraint over the last three decades.

## Introduction

Water is fundamental for plant growth, and vegetation response to water availability influences water, carbon, and energy exchanges between land and atmosphere^1–4^. Vegetation growth is expected to become more water constrained in a warmer climate because warming results in an increase in vapor pressure deficit and possible reductions in soil moisture^5–8^, while the observed global patterns of greening^9–11^ and increasing productivity^4,12^ may also enhance vegetation water demand. In addition, higher temperature with more frequent extreme hot days^13,14^, stronger radiation^15^, and land cover/land use changes^4^ may exacerbate water stress impacts^12,16^. Quantifying vegetation response to water availability at large spatial and temporal scales is challenging, as vegetation growth response to water availability is influenced by many interacting factors, including biome type, hydraulic strategy, water use efficiency, and location^17–21^. Simultaneously, a short-term decrease in rainfall may have both positive and negative effects on vegetation growth at different locations. For example, although water deficit negatively impacts many ecosystems, for ecosystems subjected to waterlogging or at high latitudes where temperature is a major limiting factor, short-term precipitation deficiency may result in higher temperatures, leading to enhanced vegetation growth^22–26^.

Recent studies have documented vegetation response to water availability in terms of the negative impact of drought on vegetation productivity^2,27,28^, the timescale of vegetation response to drought^18^, and vegetation resilience and recovery from severe drought^19,29^. Yet, it remains unclear whether the impact of water availability on vegetation growth is changing in a warming climate. While various water deficit impacts have been documented, to our knowledge, a comprehensive global assessment of changes in long-term vegetation response to water constraints using the full satellite record is still missing. This knowledge gap prevents an adequate understanding of vegetation response to the expected intensification of drought frequency, severity, and duration^30–35^, and change in water availability^36^. In addition, recent studies have suggested that the strength of the terrestrial carbon sink might be shifting from an increasing to a decreasing trend^6,37,38^. This is likely related to changes of water constraints on vegetation growth.

In this study, we evaluated long-term trends of vegetation response to water availability over the last three decades in the extratropical Northern Hemisphere using a robust ensemble of water availability indices and multiple indicators of vegetation growth from 1982 to 2015. We used satellite-derived normalized difference vegetation index (NDVI), enhanced vegetation index (EVI), vegetation optical depth (VOD), solar‐induced chlorophyll fluorescence (SIF), and gross primary productivity (GPP) as proxies of vegetation growth; and self-calibrating Palmer drought severity index (scPDSI) and Standardized Precipitation Evapotranspiration Index (SPEI) aggregated over a range of time-scales as proxies of water availability. Both SPEI and scPDSI are meteorological water availability indices, and high to low values signify relatively wet to dry conditions in a given area as compared to its long-term average. Therefore, a year with low SPEI and scPDSI in a wet area may not necessarily cause vegetation water stress and may still be wetter than a year with high SPEI and scPDSI in a dry region^39^. The statistical relationship between water availability indices and vegetation growth has been widely applied to examine the response of vegetation growth to water availability^18,40–42^.

## Results and discussion

### Metrics to quantify the changes in water deficit, water surplus, and vegetation response time

**Fig. 1:**
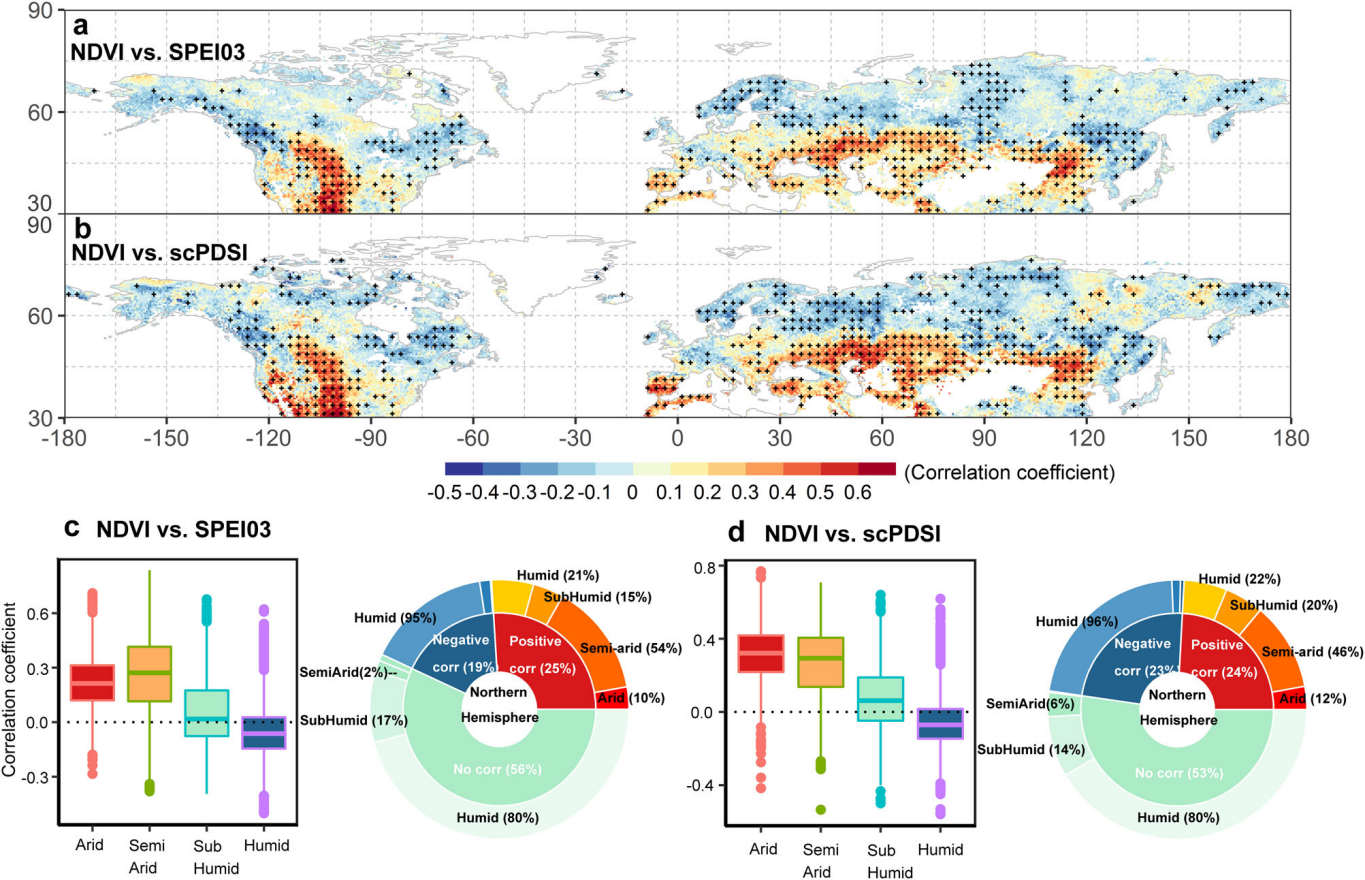
Spatial distribution of the correlations between vegetation growth and water availability indices over the last three decades. **a**, **b** Show the spatial distribution of correlation coefficients (R_NDVI-SPEI03_ and R_NDVI-scPDSI_) between normalized difference vegetation index (NDVI) anomaly and 3-month Standardized Precipitation-Evapotranspiration Index (SPEI03) and Palmer Drought Severity Index (scPDSI) for the entire study period. Black dots indicate significant Spearman correlations with *p* < 0.05; **c**, **d** are the statistical distributions of R_NDVI-SPEI03_ and R_NDVI-scPDSI_ for arid, semi-arid, sub-humid, and humid regions, respectively. The maximum and minimum extents of the colored boxes indicate the 25^th^ and 75^th^ percentiles and the whiskers represent the 5^th^ and 95^th^ percentiles, respectively.

Here we evaluated the relationship between:1) vegetation growth and SPEIs with time-scales ranging from 1- to 24-months; and 2) vegetation growth and scPDSI over a fixed time-scale. SPEIs calculated over a specific time-scale represent the cumulative water balance over the total months of the specified time-period (e.g., SPEI03 represents the 3-month cumulative water balance)^18,39^. A significant positive correlation between NDVI and SPEI/scPDSI (*p* < 0.05) means that NDVI is increasing with wetting and decreasing with drying, suggesting that in the areas where this happens, vegetation growth is constrained by water scarcity. We term the area associated with significant positive correlation between NDVI and SPEI/scPDSI (*p* < 0.05) as “vegetation water deficit regions”. In contrast, a significant negative correlation between NDVI and SPEI/scPDSI (*p* < 0.05) means that vegetation is less productive under wetter condition and more productive under drier than normal conditions, indicating a water surplus can limit vegetation growth as a result of waterlogging or of the fact that in some wetter years there might have been other factors such as temperature and solar radiation limiting productivity. We term the area associated with significant negative correlation between NDVI and SPEI/scPDSI (*p* < 0.05) as “vegetation water surplus regions”. A non-significant relationship between NDVI and SPEI/scPDSI (*p* > 0.05) indicates that vegetation growth is neither constrained by water scarcity nor by water surplus. To support the water deficit and water surplus classification based on positive and negative correlations between NDVI and SPEI/scPDSI, we showed that most of the areas with significant positive correlations between NDVI and SPEI/scPDSI (*p* < 0.05) were located in dryland regions (arid, semi-arid, and sub-humid regions) (Fig. 1), while most negative correlations were found in areas located outside drylands (or “non-dryland” regions, Fig. 1) (see “Methods” for dryland vs. non-dryland definitions by aridity index). Specifically, humid regions accounted for 95% and 96%, respectively, of the significant negative R_NDVI-SPEI03_ (i.e., *r*-value between NDVI and 3-month time-scale SPEI, namely SPEI03; *p* < 0.05) and R_NDVI-scPDSI_ (i.e., *r*-value between NDVI and scPDSI; *p* < 0.05) (Fig. 1c and d). By contrast, drylands accounted for 79% and 78% of the significant positive R_NDVI-SPEI03_ and R_NDVI-scPDSI_ (Fig. 1c and d). Moreover, the vast majority of semi-arid and arid regions were associated with significant positive R_NDVI-SPEI03_ (87% and 84%, respectively) and R_NDVI-scPDSI_ (88% and 89%, respectively) (Supplementary Fig. 1). This result is consistent with the expectation that most drylands are water-limited, while only some humid regions, mainly characterized by peatlands, bogs, and other wetlands where waterlogging has been reported to limit productivity^43–45^, experience water surplus. A time lag typically exists between the onset of water scarcity and the emergence of observable consequences on vegetation^18^. Therefore, we defined the minimum vegetation response time to water stress (“response time” hereafter) as the minimum lag time between a drop in water availability (i.e., the onset of drier conditions) and the first observed impact on vegetation productivity. A shorter response time suggests a stronger susceptibility of vegetation to water stress. Similarly, we also defined the maximum water surplus period (“water surplus period” hereafter) as the maximum time between the start of drying and the last observed positive effect on vegetation growth. Shorter water surplus periods correspond to shorter periods in which vegetation growth experiences water surplus. The response time and water surplus period metrics allow us to quantify changes in the time of vegetation response to changes in water availability. We quantitatively attributed the observed changing responses of vegetation growth to water availability to multiple meteorological factors and atmospheric CO_2_ concentrations using attribution analysis.

### Observed increasing water constraint on vegetation growth

**Fig. 2:**
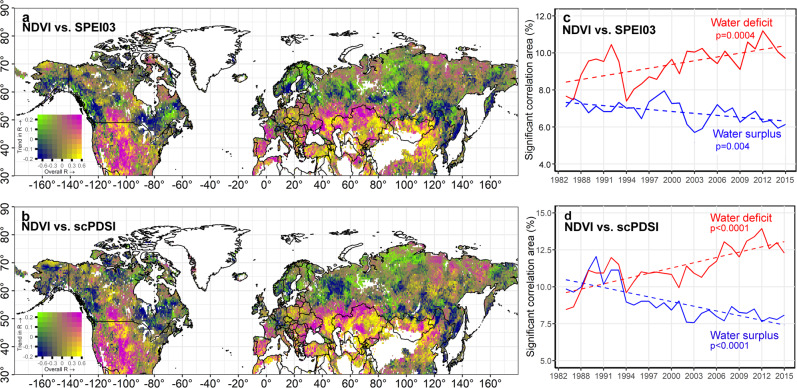
Spatiotemporal distribution of the statistically significant correlations between vegetation growth and water availability indices over the last three decades. **a**, **b** Show distribution of correlation coefficients (R_NDVI-SPEI03_ and R_NDVI-scPDSI_) between normalized difference vegetation index (NDVI) anomaly and 3-month Standardized Precipitation-Evapotranspiration Index (SPEI03) and Palmer Drought Severity Index (scPDSI). The horizontal axis of the color legend is the correlation coefficient between NDVI anomaly and SPEI03 (scPDSI) for the entire study period, the vertical axis of the color legend is the trend of correlation coefficient for the 30 five-year moving windows, no color indicates unvegetated regions. The chartreuse color stands for vegetation water surplus regions where water surplus has been decreasing; navy color indicates vegetation water surplus regions that have been experiencing an increase in water surplus; magenta color is used for water deficit regions that have been seeing an increase in water deficit; and regions colored in yellow are characterized by water deficit and a decrease in water deficit. **c**, **d** Show the temporal trends of significant changes in percentage areas associated with water deficit and water surplus responses using 5-year moving windows. Blue color stands for the water surplus response and red color for water deficit response. All the trends of water deficit and water surplus responses are significant in linear trend test and Mann–Kendall trend test (*p* < 0.05). X-axes of (**c**, **d**) are binned using 5-year moving window to smooth out time series fluctuations and highlight the trends.

Multiple lines of evidence show a markedly increasing water constraint on extratropical Northern Hemisphere vegetation growth over the last three decades, as evidenced by the expansion of water-deficit regions and the shrinking of water surplus areas over the last three decades. The spatiotemporal correlations, R_NDVI-SPEI03_ and R_NDVI-scPDSI_, indicated that most water deficit areas are located in temperate regions between 30° N and 50° N, while most water surplus regions are located in high latitude boreal regions above 50° N (Fig. 2a and b). This pattern is consistent with the fact that high-latitude boreal regions are not water limited but energy limited, and short-term precipitation deficiency may result in higher solar radiation and temperature (and in some areas less waterlogging), leading to enhanced vegetation growth^25^. We separately analyzed the trends for regions associated with water deficit and water surplus over the 1982–2015 period, and found remarkably divergent trends with a significant expansion of water deficit regions and a contraction of water surplus regions (Fig. 2c and d). The results were consistent when using either R_NDVI-SPEI03_ or R_NDVI-scPDSI_ (Fig. 2c and d).

To evaluate the robustness of our analysis, we used different moving windows, tested the impacts of long-term NDVI trends, tested the trends in sub-periods of the growing season, examined croplands separately, used different vegetation growth indicators, used water availability indicators at different time scales, and used a different water availability indicator (soil moisture) to re-conduct our spatiotemporal analysis. In addition to results based on a 5-year moving window (Fig. 2), we used 10-year and 15-year moving windows to evaluate the trends. The spatiotemporal trends in R_NDVI-SPEI03_ and R_NDVI-scPDSI_ obtained using the 10-year and 15-year moving windows (Supplementary Fig. 2) showed great consistency with those from the 5-year moving window analysis (Fig. 2a–d). To examine whether the observed trends of productivity-moisture correlations were caused by the long-term NDVI trend, we detrended NDVI using both linear and nonlinear (moving average) methods and re-conducted our analyses on spatiotemporal trends of R_NDVI-SPEI03_ and R_NDVI-scPDSI_. The results (Supplementary Fig. 3) showed great consistency with those from the non-detrended analysis (Fig. 2a–d), indicating that the observed trends of productivity-moisture correlations were not caused by the long-term NDVI trend. To examine whether the shrinking of water surplus and the increasing water deficit trends occurred in different sub-periods of the growing season, we examined the spatiotemporal productivity-moisture relationship for three sub-periods: April–June, June–August, and August–October. The temporal trends of significant changes (*p* < 0.05) in percentage areas associated with water deficit and water surplus responses for these three sub-periods (Supplementary Fig. 4) showed that the shrinking of water surplus and expansion of water deficit occurred in most of the sub-periods, except for the shrinking water surplus, which was not significant in the April–June sub-period (*p* > 0.05 for Blue line trend in Supplementary Fig. 4a and d). It is likely because the increasing snowpack melting exacerbated water surplus in the spring. Interestingly, croplands showed increasing water constraint as well, except for irrigated croplands^46^ (which only account for around 11% of all the grid cells in the study area (Supplementary Fig. 5). In addition to NDVI, we also used the VOD from 1988 to 2015, MODIS EVI, SIF, and GPP from 2000 to 2015 and GIMMS3g GPP from 1982 to 2011^47^ as vegetation growth indicators. Specifically, we evaluated to what extent the changes in the relationship between vegetation growth and water availability indices (SPEI03 and scPDSI) detected using NDVI were also consistently found with these other productivity indicators (i.e., VOD, SIF, EVI, and GPP). Our results showed comparable patterns of the changing correlations across all combinations of vegetation growth indicators and water availability indices. The trends of R_VOD-SPEI03_ and R_VOD-scPDSI _ (1982-2011), R_SIF-SPEI03_, R_EVI-SPEI03_, R_GPP-SPEI03_, R_SIF-scPDSI_, R_EVI-scPDSI_, and R_GPP-scPDSI_ (2000–2015) were all comparable to the patterns of R_NDVI-SPEI03_ and R_NDVI-scPDSI_ (1988–2015) for the overlapping periods (Supplementary Fig. 6). To support the analysis based on 3-month time-scale of SPEI (SPEI03) as water availability indicator, we re-calculated the spatiotemporal vegetation-SPEI correlations based on SPEIs at all time-scales ranging from 1 to 24 months (SPEI01-SPEI24) as water availability indicators. The significant negative and positive trends in the correlation between NDVI anomaly and SPEI (p < 0.05) were confirmed at all time-scales ranging from 1 to 24 months (Supplementary Fig. 7). In addition to SPEI and scPDSI, we also used European Space Agency (ESA) Climate Change Initiative (CCI) program based soil moisture as water availability indicator to evaluate the robustness of spatiotemporal trends of R_NDVI-SPEI03_ and R_NDVI-scPDSI_. The spatiotemporal relationship between vegetation growth and soil moisture (R_NDVI-SM_) showed comparable patterns to the ones found using SPEI and scPDSI as water availability indicators (Supplementary Fig. 8).

To further support the water constraint analysis based on spatiotemporal vegetation-moisture correlations, we examined the temporal trend of NDVI anomalies under drought conditions (SPEI03 < −1.28 and scPDSI < −1) (see “Methods”). We found a significant decreasing trend (*p* < 0.05) in the drought-related NDVI z-score time series (i.e., increasing drought impact on vegetation growth) over the last three decades. The decreasing NDVI anomalies under drought (Supplementary Fig. 9) indicates an increasing drought impacts on vegetation growth, which further supports our finding that the vegetation water constraint is increasing over the last three decades.

Moreover, the mean correlation coefficient (*r*-value) between NDVI anomaly and SPEI03 across all the grid cells in the Northern Hemisphere has increased steadily over the last three decades, switching from negative to positive for the whole extratropical Northern Hemisphere (Supplementary Fig. 10). This result indicates that the extratropical Northern Hemisphere vegetation growth is becoming increasingly constrained by water deficit, in agreement with our observations that significant positive correlations (*p* < 0.05) are typically associated with water deficit while negative correlations are typically associated with water surplus (Fig. 1). The increase in atmospheric CO_2_ levels is expected to lead to higher vegetation water use efficiency^20^, and increase plant water availability, especially in drylands^48,49^. As such, CO_2_ increase would induce a more negative correlation between NDVI anomaly and SPEI03 in water deficit regions. However, this study finds a steady increase of r-value between NDVI anomaly and SPEI03 in both water deficit and water surplus regions (Supplementary Fig. 11), indicating that CO_2_ induced water saving is not sufficient to counteract the increasing water constraint.

**Fig. 3:**
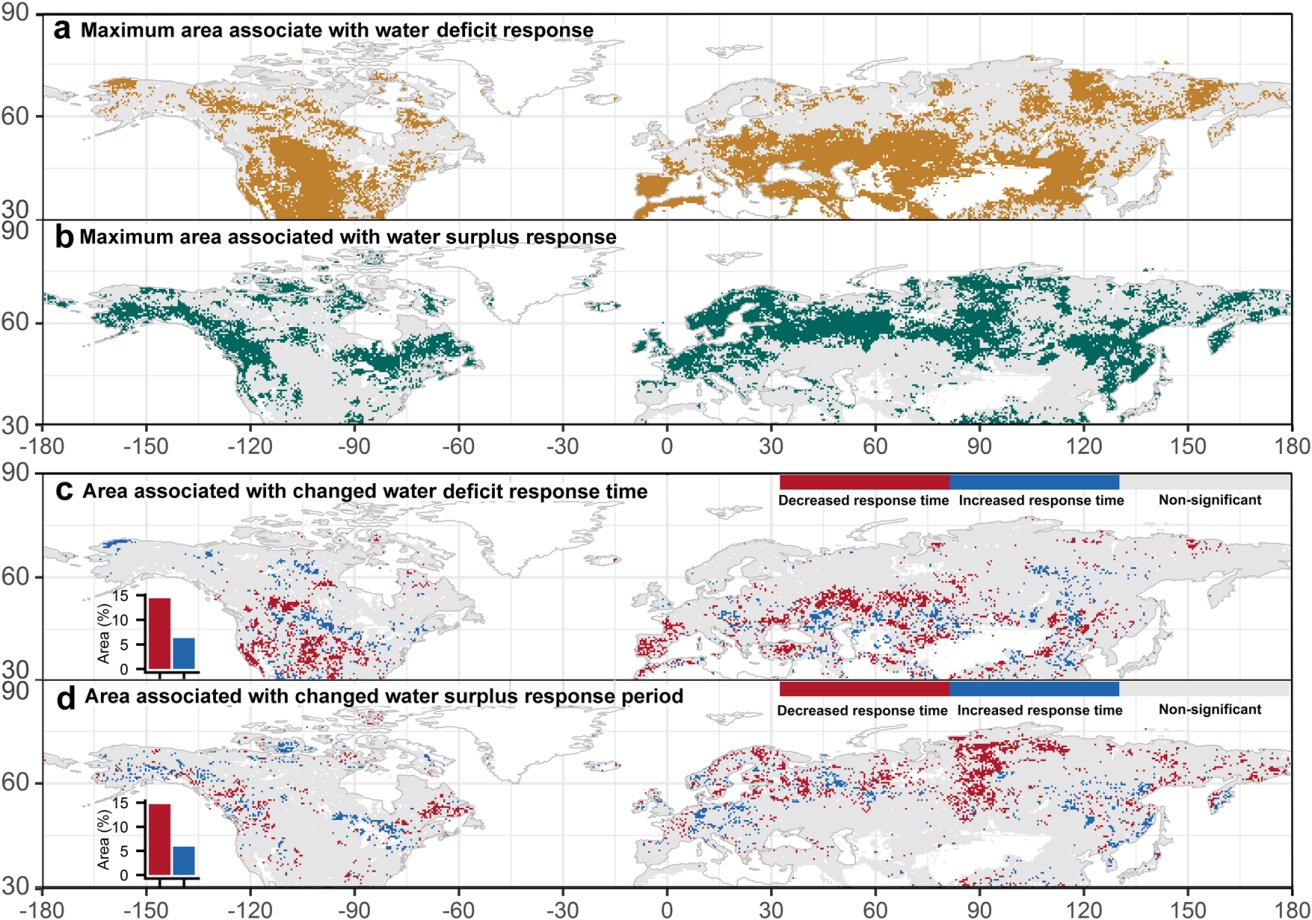
Geographical distribution of maximum areas associated with water surplus and water deficit responses as well as the areas associated with changed response times to water surplus and water deficit over the last three decades. **a** Presents the maximum area composite of significant positive correlation (water deficit response) between normalized difference vegetation index (NDVI) anomaly and Standardized Precipitation-Evapotranspiration Index (SPEIs) from 1- to 24-month time-scale (gold color); **b** the maximum area composite of significant negative correlation (water surplus response) between NDVI anomaly and SPEIs from 1- to 24-month time-scale (green color); **c** area associated with change of response time to water deficit; **d** area associated with change of response time to water surplus response. The red and blue bars in (**c**, **d**) represent the areas of decreased and increased response time, respectively.

Further, a decreasing water deficit response time and shortened water surplus period were observed between the onset of water availability change and their observable impact on vegetation across the extratropical Northern Hemisphere. We evaluated the response time and water surplus period based on the statistically significant correlations between NDVI and SPEIs at different time-scales (see “Methods”). Over the study period, 44% of the Northern Hemisphere regions had NDVI positively correlated to at least one time-scale of SPEIs (Fig. 3a) and 38% of the Northern Hemisphere regions had NDVI negatively correlated to at least one time-scale of SPEIs ranging from 1 to 24 months (Fig. 3b). This indicates that 44% of the Northern Hemisphere regions can be considered water deficit region and 38% of the Northern Hemisphere regions can be considered water surplus region for at least one month over the last 30 years. More importantly, our analysis indicates that about 14% of the Northern Hemisphere land area showed a significant decrease (*p* < 0.05) in response time, whereas 6% showed a significant increase (*p* < 0.05) in response time, resulting in an overall expansion of regions with decreased water deficit response time. In other words, there is an expansion of regions exhibiting a shorter water deficit response time, which corresponds to an increased vegetation susceptibility to stress induced by water scarcity (Fig. 3c). Similarly, about 6% of the Northern Hemisphere regions showed an increased water surplus period while 15% of the regions showed a decreased water surplus period, resulting in an overall expansion of regions with decreased water surplus period (i.e., shortened water surplus period) (Fig. 3d). Based on the previous results, a shorter water surplus duration is expected to favor plant productivity in water surplus regions. The shorter water deficit response time and shortened water surplus period across the Northern Hemisphere are also in support of the notion that vegetation growth in the Northern Hemisphere over the last three decades has become increasingly limited by water scarcity and not by surplus.

**Fig. 4:**
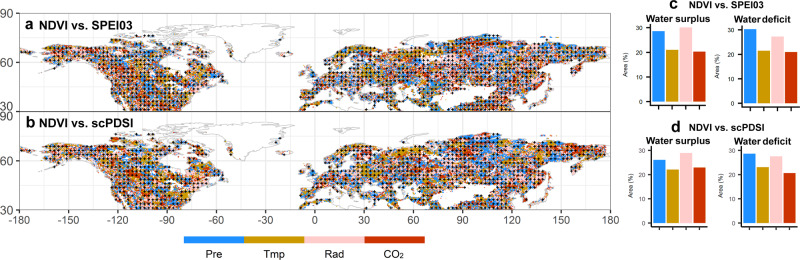
Attribution of meteorological factors and atmospheric CO_2_ to the correlations between normalized difference vegetation index (NDVI) anomaly and water availability indices over the last three decades. **a**, **b** are the spatial distributions of the dominant factor influencing R_NDVI-SPEI03_ and R_NDVI-scPDSI_, respectively. The dots show the regions that R_NDVI-SPEI03_ or R_NDVI-scPDSI_ variations are significantly explained by precipitation, radiation, temperature, and atmospheric CO_2_. **c**, **d** The percentage areas where the water deficit and water surplus responses can be explained by one of the four dominant factors (i.e., precipitation, temperature, radiation, and CO_2_). Pre: precipitation, Rad: radiation, Tmp: temperature, CO_2_ : atmospheric CO_2_.

We finally evaluated the role of air temperature, precipitation, solar radiation, and atmospheric CO_2_ in mediating vegetation responses to water availability by carrying out an attribution analysis. We applied a partial correlation algorithm (see “Methods”) to attribute the R_NDVI-SPEI03_ and R_NDVI-scPDSI_ to meteorological and atmospheric CO_2_ drivers (i.e., air temperature, precipitation, solar radiation, and atmospheric CO_2_). The partial correlation of each factor was calculated for each grid cell (Supplementary Fig. 12). The factor associated with the largest absolute value of partial correlation was identified as the dominant factor to the R_NDVI-SPEI03_ and R_NDVI-scPDSI_ in that grid cell (Fig. 4a and b). The area associated with each dominant factor was then calculated for the water deficit and the water surplus regions separately (Fig. 4c and d). The area fraction associated with each dominant factor was calculated for the water deficit and the water surplus regions, respectively (i.e., the dotted blue and red color regions as identified in Fig. 1a and b). While the partial correlation analysis revealed that there was no single driver responsible for the increasing vegetation water constraint, R_NDVI-SPEI03_ and R_NDVI-scPDSI_ were attributable to precipitation and radiation for a relatively large portion of both the water deficit and water surplus regions (Fig. 4c and d). Specifically, we found that in water surplus regions (i.e., the dotted blue regions in Fig. 1a), 29% and 30% of R_NDVI-SPEI03_ was attributable to precipitation and radiation, respectively (Fig. 4c). Similarly, in water deficit regions (i.e., the dotted red color regions in Fig. 1a), 30% and 27% of R_NDVI-SPEI03_ was attributable to precipitation and radiation, respectively (Fig. 4c). The areas where R_NDVI-scPDSI_ was explained by precipitation and radiation were consistent with those for R_NDVI-SPEI03_ (Fig. 4c and d). It was also found that in water surplus regions 21% and 20% of the R_NDVI-SPEI03_, and 22% and 22% of the R_NDVI-scPDSI_ were attributable to temperature and CO_2_, respectively (Fig. 4c and d). In water deficit regions, 21% and 21% of R_NDVI-SPEI03_, and 23% and 21% of R_NDVI-scPDSI_ were attributable to temperature and CO_2_, respectively (Fig. 4c and d). The most likely explanation that the observed productivity-moisture correlations were attributable to a greater extent to precipitation and radiation in both water deficit and water surplus regions is that these two meteorological variables capture water and energy constraints and are closely related at large spatial scales. For example, most of the water deficit regions (e.g., the regions in the south and west of the United States) showed decreasing precipitation and slightly increasing radiation (Supplementary Fig. 13a and b); whereas water surplus regions (e.g., southeast United States and multiple regions in Russia) showed increased shortwave radiation and to a lesser extent decreasing precipitation (Supplementary Fig. 13a and b). The fact that in some regions temperature is the major attribution factor for the observed correlation patterns is likely due to the increasing frequency in extreme hot days^14^, which in turn are associated with higher atmospheric water demand^6^. In high-latitude water surplus regions, plants affected by cell damage caused by more frequent extreme hot days may consistently experience reduced growth even in drier periods. In water deficit regions higher temperature and solar radiation increase evapotranspiration/potential evapotranspiration making the soils drier, exacerbating vegetation water stress exposure^15,50^. For the regions where R_NDVI-SPEI03_ and R_NDVI-scPDSI_ are dominated by CO_2_, we may be capturing an increase in vegetation water use efficiency under increasing CO_2_ concentrations^20^. To evaluate the robustness of our attribution analysis, we also used a “relaimpo”^51^ relative importance analysis to quantify the relative contributions of each factor. Results from the relative importance analysis were comparable to those from the partial correlation analysis (Supplementary Fig. 14).

These results, which appear to be robust with respect to possible trends induced by sensor aging/shift and other sources of uncertainty such as drought legacy effect and grid cell contaminations (see “Methods”), provide multiple lines of evidence for an overall increase of water constraints on extratropical Northern Hemisphere vegetation growth over the last 30 years. With future warming, regions experiencing water constraints will likely increase, resulting in a reduction of carbon uptake across the extratropical Northern Hemisphere, thus potentially amplifying the global carbon-climate feedback. Consistent with previous studies^23,37,38^, we found that the increasing vegetation water constraints are associated with increasing negative drought impacts hidden in the overall greening trends, with plants becoming increasingly sensitive to drought. With the projected increasing frequency and severity of drought events^31^, the increasing water constraint is likely to be the one major factor causing the strength of the terrestrial carbon sink to shift from an increasing to a decreasing trend under climate change. More importantly, even without drought, the increasing vegetation water constraint could be one of the main factors counteracting the fertilization effect from CO_2_ and nitrogen on vegetation growth, leading to a decreasing trend of terrestrial carbon sink under the warming climate.

## Methods

### Satellite observation data

The third-generation biweekly Advanced Very High Resolution Radiometer (AVHRR) NDVI (GIMMS-NDVI3g, available at https://ecocast.arc.nasa.gov/data/pub/gimms/3g.v0) data were used in this study as a proxy for vegetation growth between 1982 and 2015. The study period of 1982–2015 was selected to balance the data availability for vegetation growth and water availability indicators. For example, the Standardized Precipitation-Evapotranspiration Index (SPEI) dataset is currently available only to the year 2015. The GIMMS-NDVI3g data have corrections for sensor degradation, cloud cover, inter-sensor differences, solar zenith angle, viewing angle effects, and volcanic aerosols, making them widely used to study the vegetation dynamics under warming climate^4,13^. Ku-band vegetation optical depth (VOD) was selected from 1988 to 2015. The maximum value composite method was applied to composite daily VOD into monthly datasets, and the cubic resampling method was used to aggregate 0.05° spatial resolution into 0.5° to match the spatial resolution from climate datasets. Daily Ku-band VOD data^52^ were obtained from https://zenodo.org/record/2575599 #.XzivuMBKipp. Moderate Resolution Imaging Spectroradiometer (MODIS) based enhanced vegetation index (EVI) and gross primary production (GPP), as well as solar-induced chlorophyll fluorescence (SIF) data derived from discrete the Orbiting Carbon Observatory-2 (OCO-2) SIF and MODIS observations (GOSIF)^53^, were selected from 2000 to 2015 as complementary proxies for vegetation growth and were used here for evaluating the robustness of the observed trends based on NDVI. GOSIF uses a combination of the OCO-2 SIF with MODIS version 6 data, which alleviated the issue of sensor degradation. Monthly GOSIF data were obtained from http://data.globalecology.unh.edu/data/GOSIF/. Version 6 of EVI (MOD13A3) and GPP (MOD17A2H) were aggregated to 0.5° × 0.5° to match the resolution of meteorological data. In addition, GPP data from 1982 to 2011 based on MODIS GPP algorithm driven by GIMMS fraction vegetation absorbed photosynthetically active radiation (fPAR) and leaf area index (LAI) data^47^ were used to evaluate the robustness of the observed trends using EVI and GPP and to make up for the absence of MODIS-based vegetation growth data before 2000. The maximum value composite method and the cubic resampling method were used to composite 8-day and bi-weekly data into monthly with 0.5° spatial resolution for the remote sensing-based data to match the spatial and temporal resolution of water availability indices and climate data^13^. The detailed evaluation of MODIS algorithm based GIMMS-GPP from 1982 to 2011 could be found in Smith et al.^47^.

### Gridded water availability indices

Standardized Precipitation-Evapotranspiration Index (SPEI) and self-calibrating Palmer Drought Severity Index (scPDSI) are widely used as water availability indices to quantify water deficit onset, duration, magnitude, and spatial extent^5,19,29,34^. The monthly scPDSI, calculated based on balance model of precipitation, temperature, and potential evapotranspiration (PET)^54,55^ were provided by the Research Data Archive at the National Center for Atmospheric Research (NCAR). The monthly scPDSI was aggregated to 0.5° × 0.5° using cubic resampling method. Monthly SPEI was calculated based on historical probability distribution of precipitation minus potential evapotranspiration (PET)^39^. Both SPEI and scPDSI used Penman-Monteith method to calculate PET. The main difference between SPEI and scPDSI is that SPEI provides multiple time-scales and scPDSI is with fixed time-scale^39^. The 0.5° with 1–24 time-scales of SPEI were selected in this study to characterize the cumulative water balance conditions from the previous 1–24 months^39^. 1–24 month time-scale of SPEIs were obtained from http://digital.csic.es/handle/10261/153475, and scPDSI from 1982 to 2015 are available at https://rda.ucar.edu/datasets/ds299.0/. Aridity index (AI) is defined as the ratio of mean annual rainfall to mean annual potential evapotranspiration. Aridity index was used identify arid (AI < 0.2), semi-arid (0.2 ≤ AI ≤ 0.5), sub-humid (0.5 ≤ AI ≤ 0.65), and humid (AI ≥ 0.65) regions^56,57^. The spatial AI dataset was obtained from https://cgiarcsi.community/data/global-aridity-and-pet-database/. The 30 arc seconds spatial resolution was aggregated to 0.5° × 0.5° using cubic resampling method to match the resolution of water availability indices and meteorological data. In addition to SPEI and scPDSI, Essential Climate Variable (ESA) soil moisture data (version 05.2)^58–60^ (www.esa-soilmoisture-cci.org/) was used as an additional water availability indicator to evaluate the water constraint and water surplus trends. The daily ESA soil moisture data were aggregated into monthly using monthly mean values.

### Forcing datasets

Monthly meteorological (including air temperature, precipitation, incoming shortwave radiation) and atmospheric CO_2_ data with spatial resolution of 0.5° × 0.5° were used to quantify the attributions of the observed relationships between vegetation growth and water availability indices from 1982 to 2015. Monthly air temperature and precipitation data with a spatial resolution of 0.5° × 0.5° were obtained from Climate Research Unit (CRU) at the University of East Anglia (CRU TS 3.23)^61^. The shortwave radiation data was obtained from the Terrestrial Hydrology Research Group at Princeton University^62^ (http://hydrology.princeton.edu/data/pgf/v2/0.5deg/monthly/).

### Trend analyses of vegetation and water availability relationships

To examine the general change of vegetation growth responses to water availability, we conducted the analyses of the spatiotemporal relationship between the growing season (April to October) NDVI anomaly and the two water availability indices (SPEI03 and scPDSI) for each grid cell over the Northern Hemisphere from 1982 to 2015. SPEI03 represents 3-month Standardized Precipitation-Evapotranspiration Index. NDVI anomaly was used to remove the effect of seasonality, and we calculated the NDVI anomaly based on z-score of NDVI using the formula of ${A}_{j,i}=(\frac{{{NDVI}}_{j,i}-\bar{{{NDVI}}_{j}}}{\sigma })$, where ${A}_{j,i}$ denotes NDVI anomaly for the month *j* in year *i*, $\bar{{{NDVI}}_{j}}\,$denotes the averaged NDVI of month j over 1982–2015; $\sigma$ stands for the standard deviation of NDVI for month *j* over 1982–2015. Spearman rank correlation coefficients (*r*-values) between NDVI anomaly and the two water availability indices (R_NDVI-SPEI_ and R_NDVI-scPDSI_) were used to represent the NDVI and SPEI (or scPDSI) relationship. Grid cells with significant positive and negative correlations (*p* < 0.05) between vegetation indicators and water availability indices were defined as grid cells associated with water deficit and water surplus, respectively. The ratio between the sum of all the grid cells associated with water deficit and total grid cells was defined as the percentage area associated with water deficit across the Northern Hemisphere each year. The same approach was used to calculate the percentage area associated with water surplus across the Northern Hemisphere each year. Given that the time-scales at which different biome types respond to water availability may differ noticeably^19^, in addition to SPEI03, we further estimated the correlations between NDVI anomaly and SPEI separately for 1 to 24 month time-scales. In addition to SPEI and scPDSI, ESA soil moisture data was used as one additional water availability indicator and spatiotemporal relationships between the growing season (April to October) NDVI anomaly and ESA soil moisture were also conducted.

To quantify the spatiotemporal dynamics of water deficit and water surplus regions over the last three decades, the trends of correlation coefficients between vegetation growth indicators and water availability indices of 5-year moving window for each grid cell were analyzed using linear and Mann–Kendall trend test (e.g., Fig. 2a and b). We used 5-year moving window in our trends analysis in this study to smooth out time series fluctuations and highlight trends. Ten-year and 15-year moving window also used in our study to evaluate the robustness of the 5-year moving window trend analysis. A longer the moving window resulted in less total time series points and a reduction in fluctuations from time point to time point, which helped to highlight any potential long-term trend. We focus our presentation of the results on the 5-year moving window analysis since it maximized the number of time series points while still consistently highlighting any emergent long-term trends in the data. To analyze the changes in areas associated with water deficit/water surplus across the Northern Hemisphere, the trend of percentage area associated with water deficit/water surplus was analyzed with a 5-year moving window using linear and Mann–Kendall trend analysis (e.g., Fig. 2c and d). To evaluate the robustness of the relationships between NDVI and the two water availability indices, we used the same method to analyze the relationships between VOD, SIF, EVI, and GPP and the two water availability indices across the Northern Hemisphere (e.g., Supplementary Fig. 6). Temporal consistency analysis based on multiple independent satellite observations imply that the observed changes in vegetation water deficit and water surplus responses were not caused by inadequate corrections of sensors for sensor aging and sensor shift^63^. To examine whether the trends of observed productivity-moisture correlations were caused by long-term NVDI trends, we repeated the same analyses of R_NDVI-SPEI03_ and R_NDVI-scPDSI_ on detrended NDVI time series, using both linear and nonlinear detrending methods. For the linear detrending method, we first tested the linear trend significance for each pixel from 1982 to 2015 and then removed the slope of any significant increasing or decreasing trend (*p* < 0.05) in each pixel. For non-linear detrend method, we extracted the non-linear trend using interannual moving average method. The non-linear trend was extracted using the decompose function in R. We also find that the drought legacy effect (i.e., reduced vegetation growth and incomplete recovery after extreme drought events)^64,65^ is unlikely to play a dominant role in influencing the trend of vegetation response to water availability since we found similar trends in NDVI responses to 1 month SPEI to 24 months SPEI (Supplementary Fig. 7). Nevertheless, multiple uncertainties may affect the understanding of the water constraint changes on vegetation in our study. These may include uncertainties of water availability and vegetation indices at high latitudes, uncertainties of vegetation growth indicators due to snow and/or cloud contamination, and uncertainties due to unaccounted for insect and fire disturbances^66^. However, because we found multiple lines of evidence of increasing vegetation water constraint, we do not expect these uncertainties to affect our key findings.

To further test the vegetation water constraint trends in addition to spatiotemporal vegetation-moisture correlations, we examined the temporal trend of NDVI anomalies under drought. To examine the drought impacts on vegetation while minimize impacts from other confounding factors such as CO_2_ fertilization and lengthening of the growing season, we used both linear and nonlinear detrending methods for each grid cell. Linear detrending consisted in removing the slope of monthly NDVI for five years intervals between 1982 and 2015. Nonlinear detrending consisted in removing the moving average of interannual trend. The de-trended NDVI anomaly was then calculated based on the de-trended z-score of growing-season (April–October) NDVI. We then analyzed the change of de-trended NDVI anomaly under drought conditions. We extracted all the grid cells of de-trended NDVI anomaly that are under drought conditions indicated by 3-month time-scale SPEI (SPEI03) and scPDSI from 1982 to 2015. scPDSI < −1 and SPEI03 < −1.28 were defined as drought conditions^2^.

### Analysis of vegetation response time to water availability

It has been well documented that climate conditions accumulatively impact vegetation growth and plants have lagged response to climate over a period of time^67–69^. The concept of drought time-scale of *n* months indicates *n* months of cumulative water balance^39,70,71^ and significant relationship between NDVI and *n*-month time scale SPEI (*p* < 0.05) indicates the existence of a significant relationship between a *n*-month cumulative water balance indicator and vegetation growth (*p* < 0.05)^18^. Accordingly, we defined the minimum SPEI time-scale (in the 1–24 month range) associated with significant positive correlation to NDVI anomaly (*p* < 0.05) as the minimum water deficit response time for each grid cell. Similarly, we defined the maximum SPEI time-scale associated with significant negative correlation to NDVI anomaly as the maximum water surplus period (*p* < 0.05) for each grid cell. We first extracted the maximum area associated with significant vegetation water deficit by accounting for all the grid cells with significant positive correlation between NDVI anomaly and SPEIs (*p* < 0.05) in the subsequent 1- to 24- months. A given grid cell was classified as having vegetation constrained by water scarcity when at least one of the subsequent 1–24 month SPEIs exhibited a significant positive correlation with NDVI (low SPEI with low NDVI, *p* < 0.05). If in a given grid cell SPEIs had significant positive correlation with NDVI (*p* < 0.05) with more than one time lags, the minimum time-lag associated with significant positive correlation was used. Similarly, we extracted the largest areas associated with significant water surplus (*p* < 0.05) by accounting for all the grid cells with significant negative correlation between NDVI anomaly and SPEIs (*p* < 0.05) in the subsequent 1- to 24- months. A given grid cell was classified as having vegetation affected by water surplus when at least one time-lag exhibited a significant negative correlation between SPEI and subsequent NDVI (low SPEI with high NDVI, *p* < 0.05). If more than one time-lag exhibited a significant negative correlation between SPEIs and NDVI (*p* < 0.05) for a given grid cell, the maximum time-lag associated with a significant negative correlation (*p* < 0.05) was used. We then analyzed the trends of minimum water deficit response time and maximum water surplus period separately for each grid cell based on a 5-year moving window. The linear trend test was applied for each grid cell over the period of 1982–2015 to examine the significance (*p* < 0.05) of trend for vegetation response time to water availability. Since we separately examined the change of water deficit and water surplus regions, a grid with both positive and negative correlation did not affect our analysis.

### Attribution analysis

The contributions of meteorological factors (mean annual air temperature, precipitation, and shortwave radiation), and atmospheric CO_2_ to the observed trends of vegetation growth and water availability relationships were assessed using partial regression models. We fitted full models for *r*-values of the correlations between NDVI anomaly and water availability indices as a function of mean growing season air temperature, mean growing season precipitation, mean growing season shortwave radiation, and atmospheric CO_2_ of each 5-year moving window for each grid cell. We used the *p*-value (*p* < 0.05) of multivariate regression models to examine whether these factors are statistically contributed to the changes of correlations between NDVI anomaly and water availability indices (R_NDVI-SPEI_ and R_NDVI-scPDSI_). To determine the most important contributor of the R_NDVI-SPEI_ and R_NDVI-scPDSI_ temporal dynamics, we ranked these factors based on the absolute value of partial correlation coefficients of each factor. This method was applied for every grid cell of the study region to extract the most important contributor of the R_NDVI-SPEI_ and R_NDVI-scPDSI_ temporal dynamics based on the spearman partial correlation coefficient. To evaluate the robustness of the attribution analysis based on partial regression models, we also used Lindeman, Merenda and Gold (lmg) relative importance algorithm^51^ to test the relative importance of the meteorological factors and the atmospheric CO_2_ in explaining the variance of R_NDVI-SPEI_ and R_NDVI-scPDSI_. The algorithm was based on variance decomposition for multiple linear regression models; the relative importance of each factor was calculated based on variance of R_NDVI-SPEI_ and R_NDVI-scPDSI_ they explained. The relative importance was performed with the “relaimpo” package^51^ in R. This method was applied to every grid cell within the study region.

## Supplementary information

Supplementary Information

## Data Availability

The Advanced Very High Resolution Radiometer GIMMS-NDVI3g is available at https://ecocast.arc.nasa.gov/data/pub/gimms/3g.v0. 1–24 month time-scale of SPEIs were obtained from http://digital.csic.es/handle/10261/153475, and scPDSI from 1982 to 2014 is available at https://rda.ucar.edu/datasets/ds299.0/. Aridity index dataset is available at https://cgiarcsi.community/data/global-aridity-and-pet-database/. Atmospheric CO_2_ data could be available from Mauna Loa Observatory provided by the Scripps Institution of Oceanography (Scripps CO_2_ program). GIMMS-GPP from 1982 to 2011 is available at https://wkolby.org/data-code/. Monthly air temperature, shortwave radiation, and precipitation data are available at http://hydrology.princeton.edu/data/pgf/v2/0.5deg/monthly/. Monthly GOSIF data were obtained from http://data.globalecology.unh.edu/data/GOSIF/. Moderate Resolution Imaging Spectroradiometer (MODIS) based EVI and GPP datasets are available at are available from the NASA Land Processes Distributed Active Archive Center at https://lpdaac.usgs.gov. Ku-band VOD datasets are available from https://zenodo.org/record/2575599#.XyLqfLdME0M. Essential Climate Variable (ECV) soil moisture data could be obtained from www.esa-soilmoisture-cci.org/. Irrigated area extracted using Global Map of Irrigation Areas from Food and Agriculture Organization of United Nations (FAO): http://www.fao.org/aquastat/en/geospatial-information/global-maps-irrigated-areas/latest-version/.

## Code availability

All R and Python code can be available from LW upon reasonable request.
