# Supplementary material for: Observed increasing water constraint on vegetation growth over the last three decades
Source: Nat Commun. 2021 Jun 18;12:3777. doi: 10.1038/s41467-021-24016-9 (PMC8213694; doi:10.1038/s41467-021-24016-9)
Supplement: Supplementary file 1 — Supplementary Information [file 41467_2021_24016_MOESM1_ESM.pdf]

Supplementary Materials

Observed increasing water constraint on vegetation growth over the last three decades

Wenzhe Jiao[1], Lixin Wang[1]*, William K. Smith[2], Qing Chang[3], Honglang Wang[4], Paolo D'Odorico[5]

[1]Department of Earth Sciences, Indiana University-Purdue University Indianapolis, Indianapolis, IN 46202, USA.

[2]School of Natural Resources and the Environment, University of Arizona, Tucson, AZ 85719, USA.

[3]O'Neill School of Public and Environmental Affairs, Indiana University, Bloomington, IN 47405, USA.

[4]Department of Mathematical Sciences, Indiana University-Purdue University Indianapolis, Indianapolis, IN 46202, USA.

[5]Department of Environmental Sciences Policy and Management, University of California, Berkeley, CA 94720, USA.

*Correspondence to: lxwang@iupui.edu

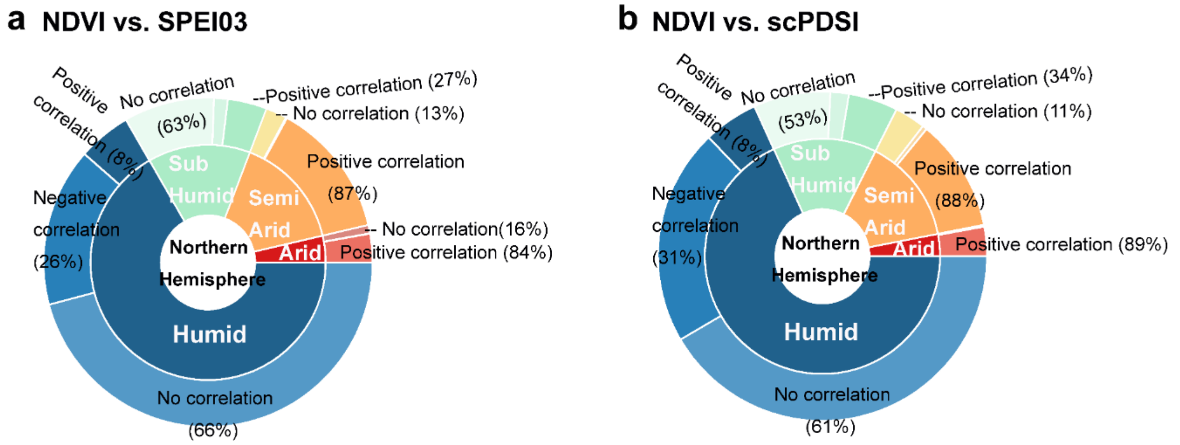

Supplementary Figure 1| The percentage of areas of the significant positive, significant negative, and non-significant correlation coefficients for arid, semi-arid, sub-humid, and humid regions, respectively between normalized difference vegetation index (NDVI) and 3-month Standardized Precipitation-Evapotranspiration Index (SPEI03) (a) and Palmer Drought Severity Index (scPDSI) (b).

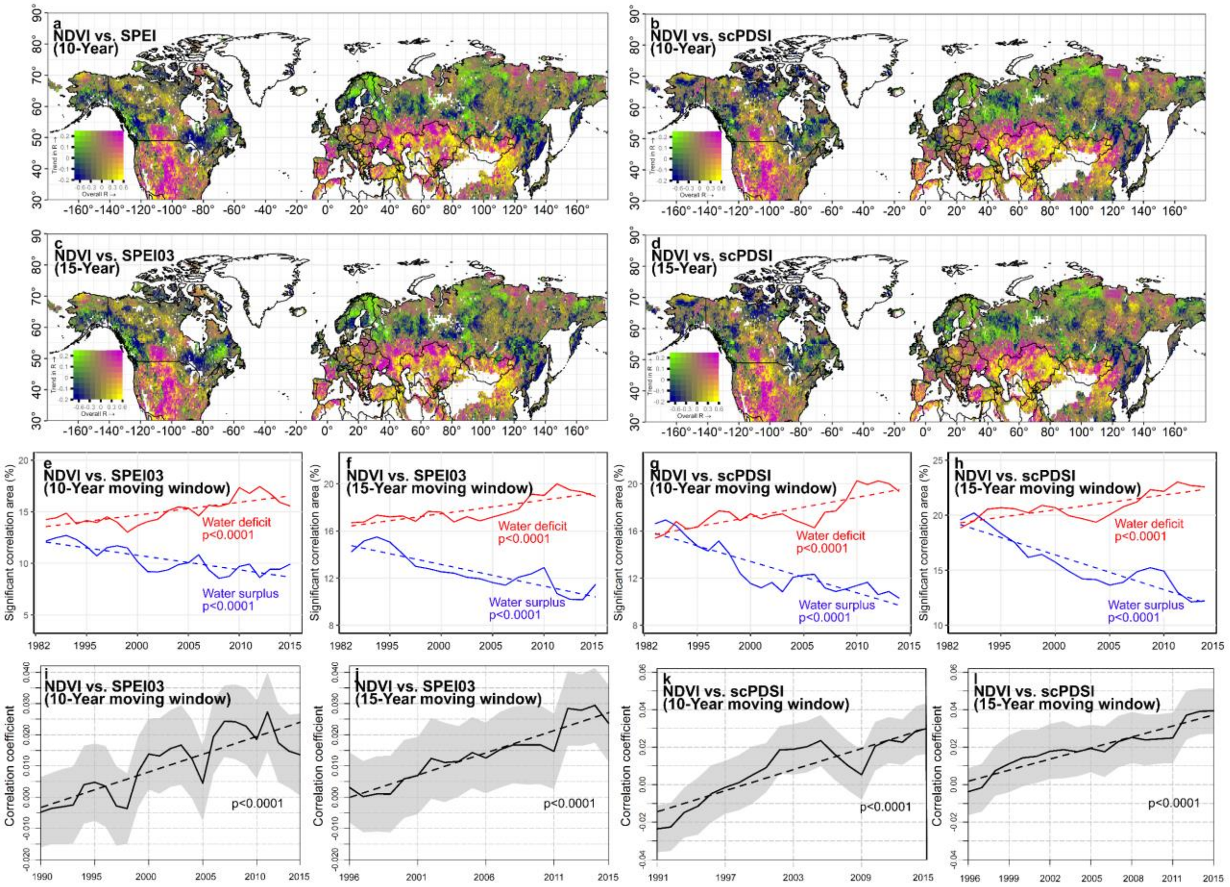

Supplementary Figure 2| Spatiotemporal distribution of the statistically significant correlations between vegetation growth and water availability indices over the last three decades analyzed using 10-year and 15-year moving window. (a) - (d) show distribution of correlation coefficients ($R_{NDVI\text{-}SPEI03}$ and $R_{NDVI\text{-}scPDSI}$) between normalized difference vegetation index (NDVI) anomaly and 3-month Standardized Precipitation-Evapotranspiration Index (SPEI) and Palmer Drought Severity Index (scPDSI) using 10-year moving window and 15-year moving window; (e) - (h) show the temporal trends of significant positive and negative correlation areas using 10-year and 15-year moving windows. Blue color stands for the negative $R_{NDVI\text{-}SPEI03}$ and $R_{NDVI\text{-}scPDSI}$ (water surplus response) and red color for positive $R_{NDVI\text{-}SPEI03}$ and $R_{NDVI\text{-}scPDSI}$ (water deficit response). (i)-(l) show the overall mean $R_{NDVI\text{-}SPEI03}$ and $R_{NDVI\text{-}scPDSI}$ over the study area using 10-year and 15-year moving windows. All the trends of positive and negative $R_{NDVI\text{-}SPEI03}$ and $R_{NDVI\text{-}scPDSI}$ are significant in linear trend test and Mann-Kendall trend test ($p < 0.05$).

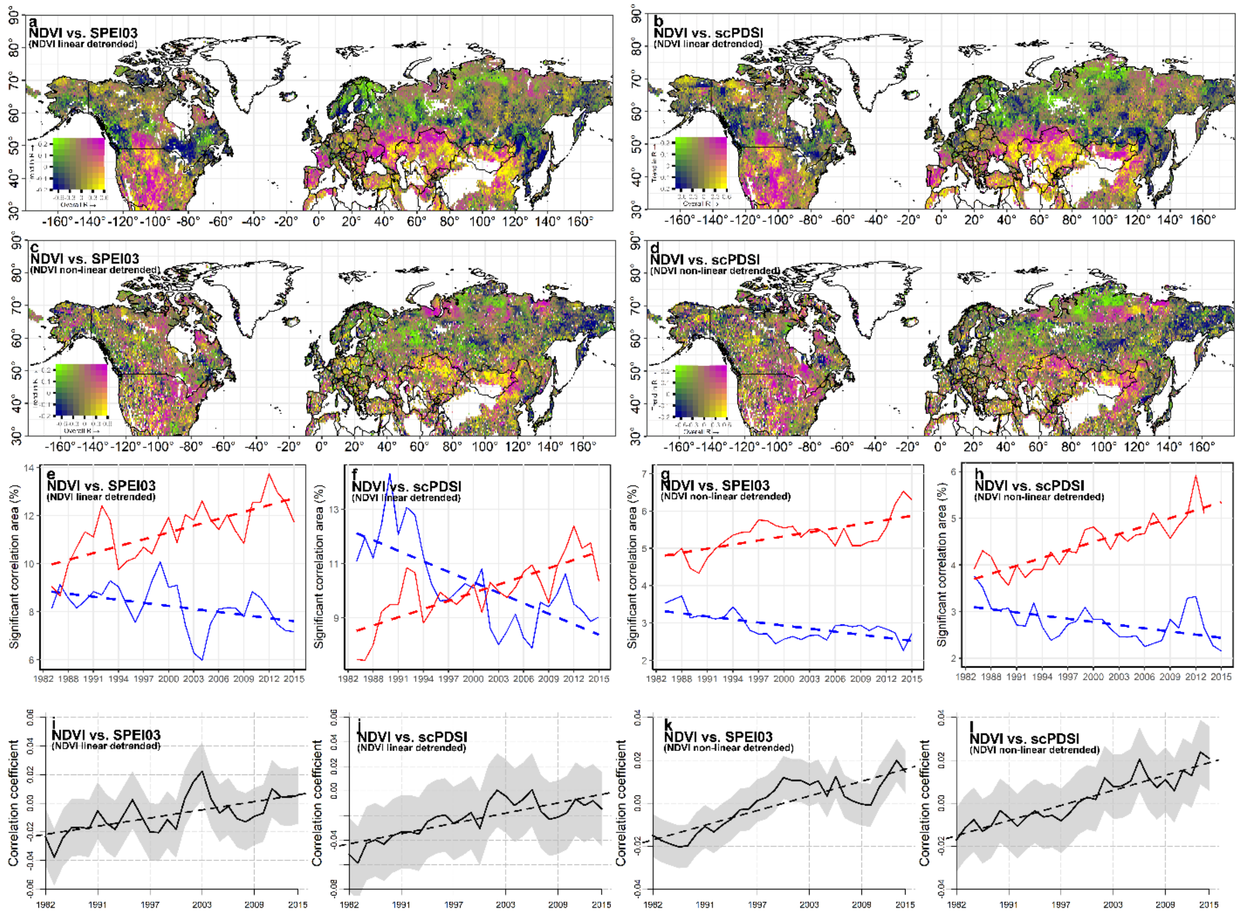

Supplementary Figure 3| Spatiotemporal distribution of the statistically significant correlations between vegetation growth and water availability indices over the last three decades analyzed after NDVI was linearly and nonlinearly detrended. (a) - (d) show distribution of correlation coefficients ($R_{NDVI-SPEI03}$ and $R_{NDVI-scPDSI}$) between normalized difference vegetation index (NDVI) anomaly and 3-month Standardized Precipitation-Evapotranspiration Index (SPEI) and Palmer Drought Severity Index (scPDSI) after NDVI was linearly and non-linearly detrended; (e) - (h) show the temporal trends of significant positive and negative correlation areas after NDVI was linearly and nonlinearly detrended. Blue color stands for the negative $R_{NDVI-SPEI03}$ and $R_{NDVI-scPDSI}$ (water surplus response) and red color for positive $R_{NDVI-SPEI03}$ and $R_{NDVI-scPDSI}$ (water deficit response). (i)-(l) show the overall mean $R_{NDVI-SPEI03}$ and $R_{NDVI-scPDSI}$ over the study area after NDVI was linear and nonlinear detrended. All the trends of positive and negative $R_{NDVI-SPEI03}$ and $R_{NDVI-scPDSI}$ are significant in linear trend test and Mann-Kendall trend test ($p < 0.05$).

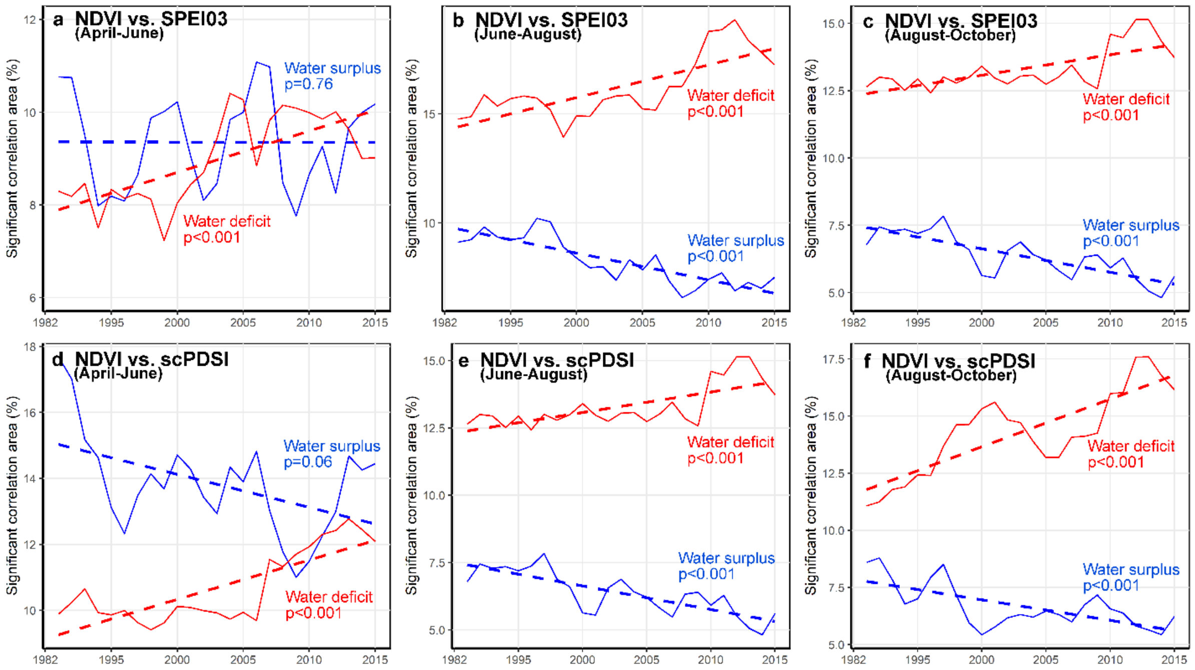

Supplementary Figure 4| Temporal trends of significant changes in percentage areas associated with water deficit and water surplus responses for sub-seasons. (a)-(c) stands for the changes of significant water surplus and water deficit areas evaluated by $R_{NDVI\text{-}SPEI03}$ for April-June (a), June-August (b), and August-October (c); (d)-(e) stands for the the changes of significant water surplus and water deficit areas evaluated by $R_{NDVI\text{-}scPDSI}$ for April-June (d), June-August (e), and August-October (f). Blue color stands for the water surplus response and red color for water deficit response.

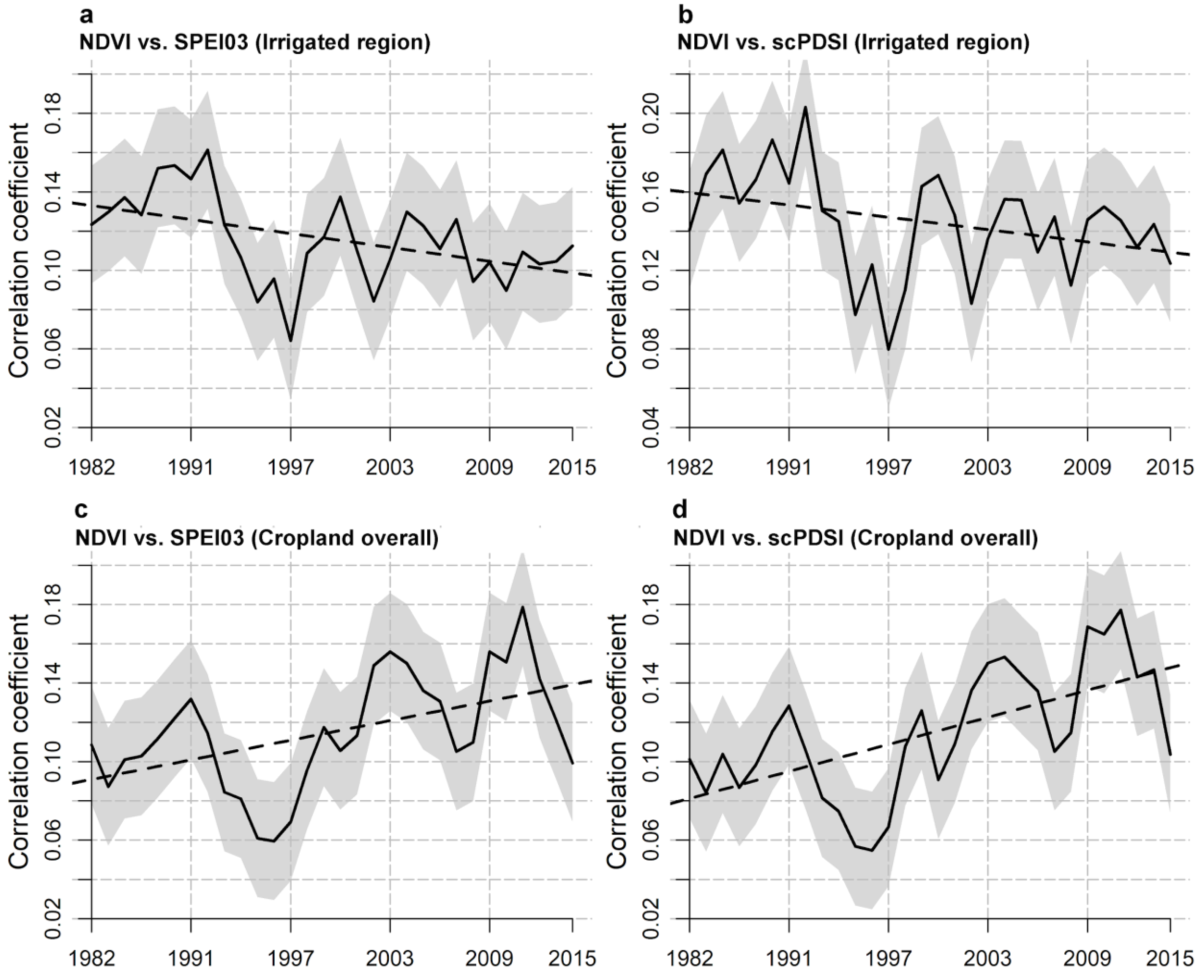

Supplementary Figure 5| The trends of mean correlation coefficients (r-value) between normalized difference vegetation index (NDVI) anomaly and water deficit indices for the irrigated cropland regions over the last three decades. (a) and (b) present r-value between NDVI and 3-month Standardized Precipitation-Evapotranspiration Index (SPEI03) and Palmer Drought Severity Index (scPDSI), respectively of all the grid cells over the irrigated cropland regions for the period of 1982-2015. Black solid lines represent the mean correlation coefficients of all the grid cells and the dashed lines represent a linear trend. The gray areas represent the means ± standard deviation of each correlation efficient. Irrigated area extracted using Global Map of Irrigation Areas from Food and Agriculture Organization of United Nations (FAO): http://www.fao.org/aquastat/en/geospatial-information/global-maps-irrigated-areas/latest version/. X-axes are binned using 5-year moving window to smooth out time series fluctuations and highlight the trends.

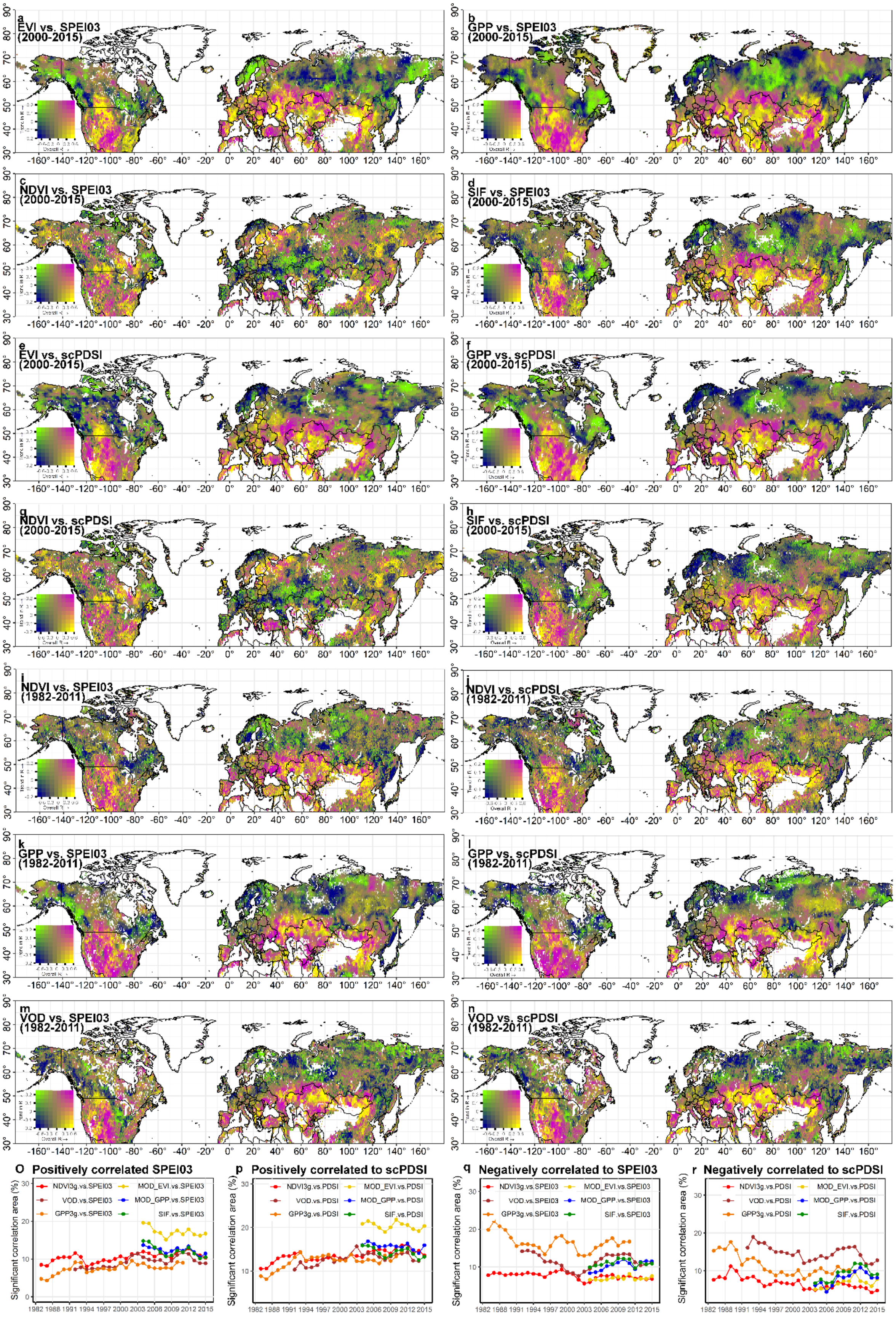
a
EVI vs. SPEI03
(2000-2015)
b
GPP vs. SPEI03
(2000-2015)
c
NDVI vs. SPEI03
(2000-2015)
d
SIF vs. SPEI03
(2000-2015)
e
EVI vs. scPDSI
(2000-2015)
f
GPP vs. scPDSI
(2000-2015)
g
NDVI vs. scPDSI
(2000-2015)
h
SIF vs. scPDSI
(2000-2015)
i
NDVI vs. SPEI03
(1982-2011)
j
NDVI vs. scPDSI
(1982-2011)
k
GPP vs. SPEI03
(1982-2011)
l
GPP vs. scPDSI
(1982-2011)
m
VOD vs. SPEI03
(1982-2011)
n
VOD vs. scPDSI
(1982-2011)
O Positively correlated SPEI03
p Positively correlated to scPDSI
q Negatively correlated to SPEI03
r Negatively correlated to scPDSI
NDVI3g.vs.SPEI03
VOD.vs.SPEI03
GPP3g.vs.SPEI03
MOD_EVI.vs.SPEI03
MOD_GPP.vs.SPEI03
SIF.vs.SPEI03
NDVI3g.vs.PDSI
VOD.vs.PDSI
GPP3g.vs.PDSI
MOD_EVI.vs.PDSI
MOD_GPP.vs.PDSI
SIF.vs.PDSI
Significant correlation area (%)

Supplementary Figure 6| Spatial distribution of the correlation coefficients (r-value) between vegetation growth and water availability indices, and the trend of significant positive and negative correlation areas over the Northern Hemisphere from 2000-2015 and 1982-2011. (a)-(h) present the spatial distribution of correlation coefficient from 2000-2015 between enhanced vegetation index (EVI) and SPEI03 (a); gross primary productivity (GPP) and SPEI03 (b); normalized difference vegetation index (NDVI) and 3-month Standardized Precipitation-Evapotranspiration Index (SPEI03) (c); solar-induced chlorophyll fluorescence (SIF) and 3-month Standardized Precipitation-Evapotranspiration Index (SPEI03) (d). EVI and Palmer Drought Severity Index (scPDSI) (e); GPP and scPDSI (f); NDVI and scPDSI (g); SIF and scPDSI (h). (i)-(l) indicate the spatial distribution of correlation coefficients from 1982-2011 between NDVI and SPEI03 (i); NDVI and scPDSI (j); GPP and SPEI03 (k); GPP and scPDSI (l); Vegetation optical depth (VOD) and SPEI03 (m); VOD and scPDSI (n). (o)-(r) show the trend of significant positive correlation area (o and p) and significant negative correlation area (q and r) between vegetation growth and water availability indices. Red color indicates the relationship between NDVI and drought indices; Brown color shows the relationship between VOD and drought indices; Orange color shows the relationship between GIMMS-GPP and drought indices; Golden color indicates the relationship between EVI and water availability indices; Blue color presents the relationship between MODIS-GPP and availability indices; Green color shows the relationship between GO-SIF and availability indices. X-axes of (o)-(r) are binned using 5-year moving window to smooth out time series fluctuations and highlight the trends.

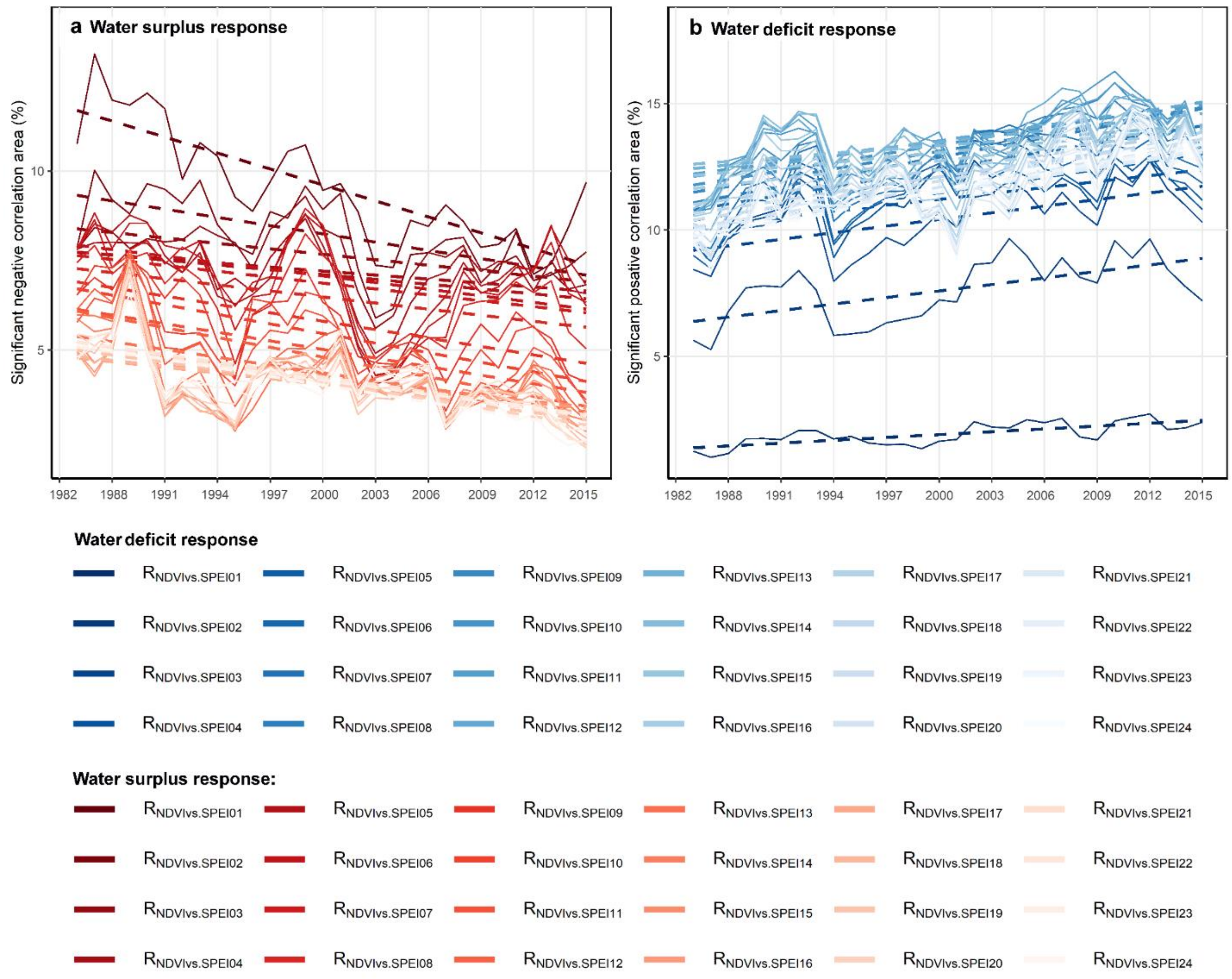

Supplementary Figure 7| Temporal trends of proportion of areas with significant correlations between vegetation growth and availability indices of different time-scales over the last three decades. (a) shows the trend of proportion of areas with significant negative correlations and (b) shows the trend of proportion of areas with significant positive correlations between normalized difference vegetation index (NDVI) and Standardized Precipitation-Evapotranspiration Index (SPEI) for all the time-scales from 1 to 24 months over the Northern Hemisphere over the period of 1982-2015. Red color is ised in the case of negative correlation and blue color is used for positive correlation. Darker color indicates shorter time-scale and lighter color indicates longer time-scale. Dashed lines represent a liner trend. All the trends are statistically significant ($p < 0.05$). X-axes are binned using 5-year moving window to smooth out time series fluctuations and highlight the trends.

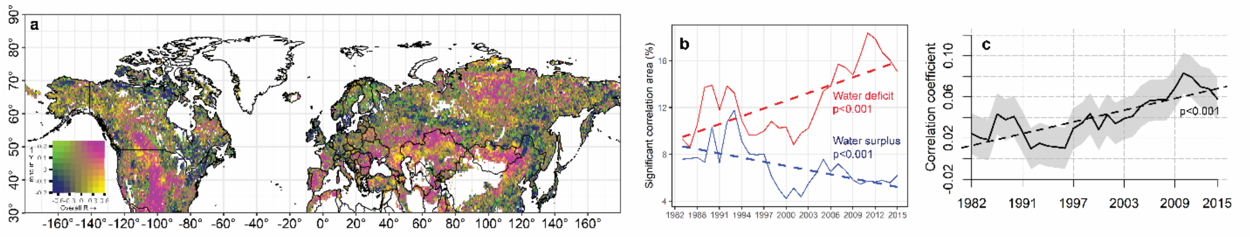

Supplementary Figure 8| Spatiotemporal distribution of the statistically significant correlations between vegetation growth and water availability indices over the last three decades: (a) shows spatial distribution of correlation coefficients ($R_{NDVI\text{-}SM}$) between normalized difference vegetation index (NDVI) anomaly and soil moisture (SM) anomaly. The horizontal axis of the color legend is the correlation coefficient between NDVI anomaly and soil moisture anomaly for the entire study period, the vertical axis of the color legend is the trend of correlation coefficient for the 30 five-year moving windows, no color indicates non-vegetation covered regions; (b) shows the temporal trends of significant changes in percentage areas associated with water deficit and water surplus responses using five-year moving windows. Blue color stands for the water surplus response and red color for water deficit response. (c) shows the overall mean correlation coefficient over the study area. Shaded areas in (c) indicates standard deviation. All the trends of water deficit and water surplus responses are significant in linear trend test and Mann-Kendall trend test ($p < 0.05$).

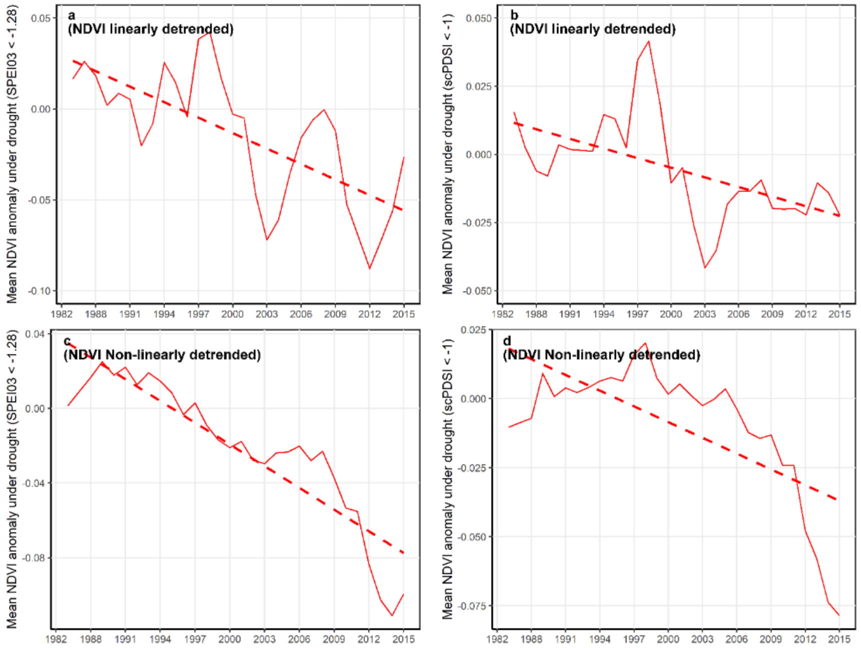

Supplementary Figure 9| Changes in de-trended normalized difference vegetation index (NDVI) anomaly under drought condition over the last three decades. Mean de-trended NDVI (linearly detrended every five years) anomaly under drought conditions indicated by 3-month (a): Standardized Precipitation-Evapotranspiration Index (SPEI03)) and (b):Palmer Drought Severity Index (scPDSI) over the Northern Hemisphere. (c)-(d) indicate mean de-trended NDVI (nonlinearly detrended based on moving average) anomaly under drought conditions indicated by 3-month (c): Standardized Precipitation-Evapotranspiration Index (SPEI03)) and (d):Palmer Drought Severity Index (scPDSI) over the Northern Hemisphere. The mean NDVI anomaly was smoothed by a five-year moving window. NDVI anomaly trend was significant in linear trend test and Mann-Kendall trend test ($p < 0.05$). Drought conditions were identified by SPEI03 less than -1.28 and scPDSI less than -1.

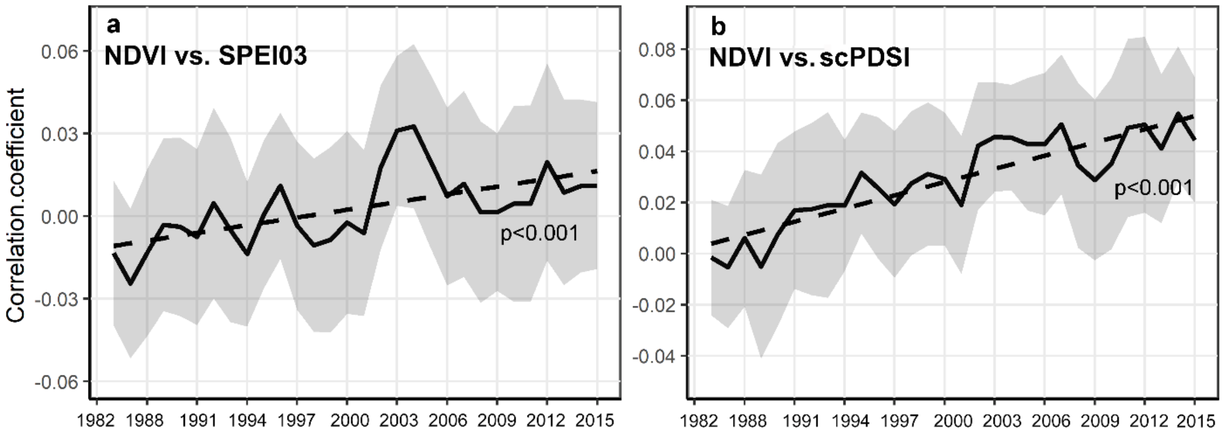

Supplementary Figure 10| The trends of mean correlation coefficients (r-value) between normalized difference vegetation index (NDVI) anomaly and water availability indices over the last three decades. (a) and (b) present r-value between NDVI and 3-month Standardized Precipitation-Evapotranspiration Index (SPEI03) and Palmer Drought Severity Index (scPDSI), respectively of all the vegetated grid cells over the Northern Hemisphere for the period of 1982-2015. Black solid lines represent the mean correlation coefficients of all the grid cells and the dashed lines represent a liner trend. The gray areas represent the means ± standard deviation of each correlation efficient. Both trends are statistically significant ($p < 0.05$). X-axes are binned using 5-year moving window to smooth out time series fluctuations and highlight the trends.

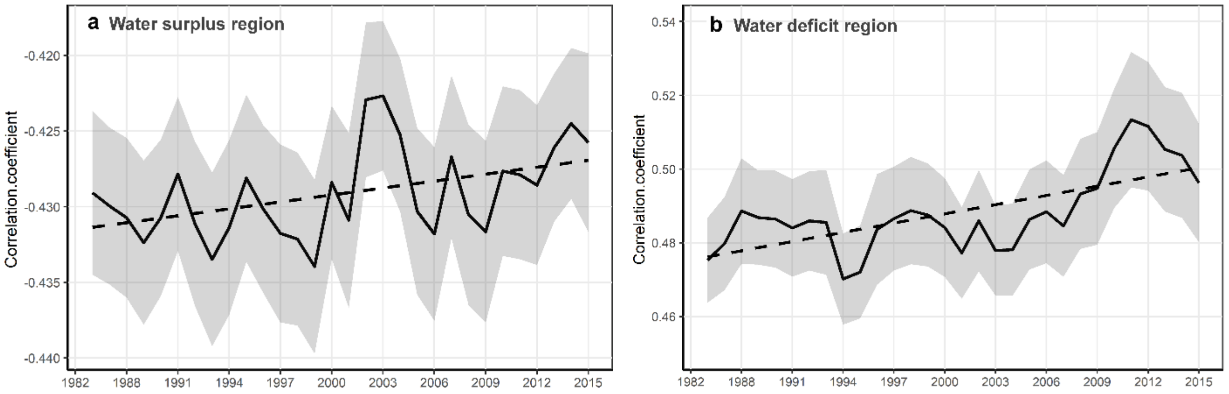

Supplementary Figure 11| The trends of mean correlation coefficients (r-value) of all the grid cells over the Northern Hemisphere for negative (a) and positive (b) correlations between normalized difference vegetation index (NDVI) anomaly and Standardized Precipitation-Evapotranspiration Index (SPEI) over the last three decades. If multiple SPEI time-scales (ranging from 1- to 24-month time-scales) were significantly correlated with NDVI for one grid cell, the minimum negative correlation coefficient for (a) or maximum positive correlation coefficient for (b) was used for this grid cell. The gray areas represent 95% confidence interval of the dashed trend line. X-axes are binned using 5-year moving window to smooth out time series fluctuations and highlight the trends. Both trends are statistically significant ($p < 0.05$).

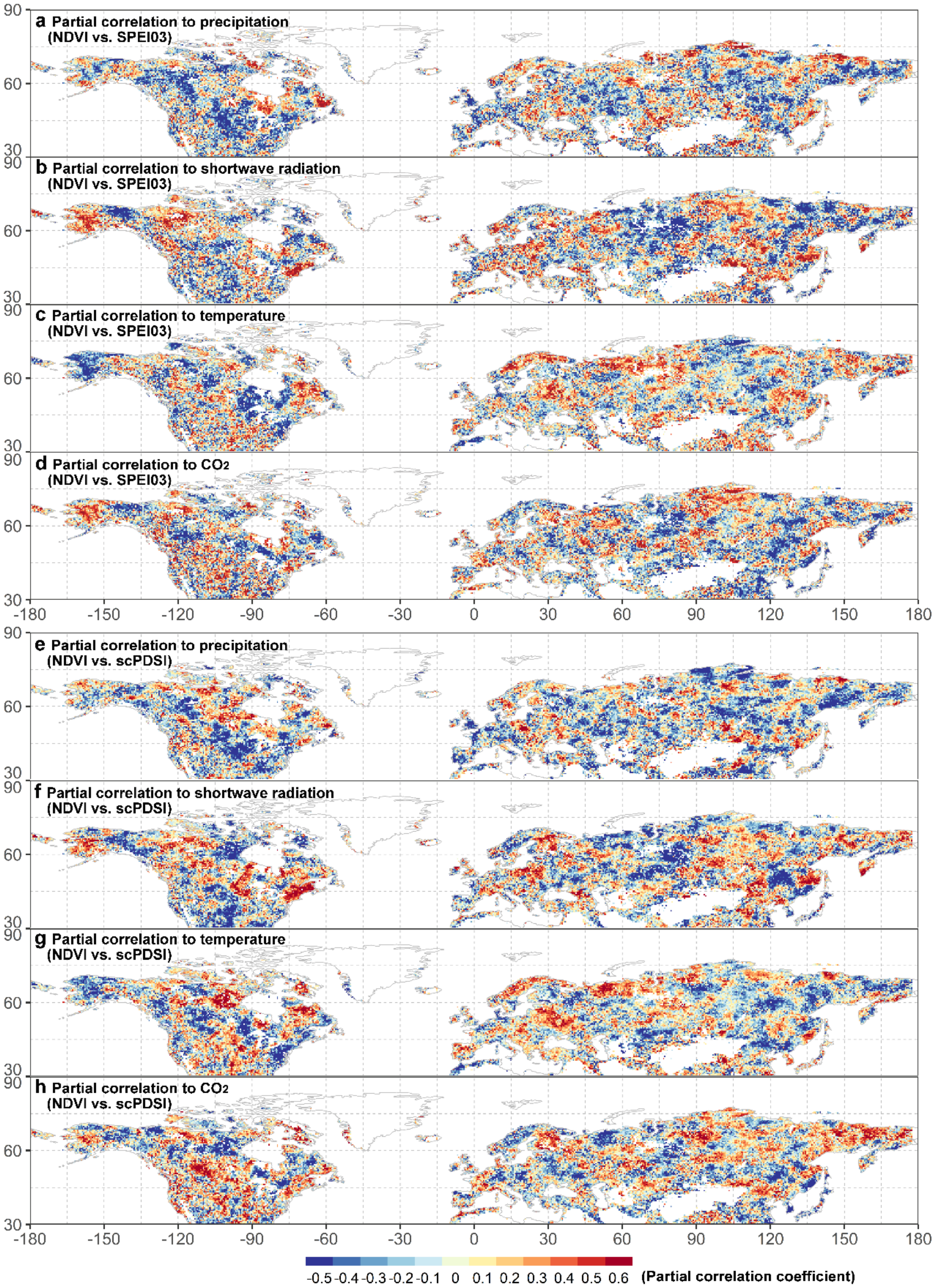

Supplementary Figure 12| Spatial distribution of partial correlation coefficient for the factors of precipitation, shortwave radiation, temperature, and atmosphere CO_2 in explaining the dynamics of $R_{NDVI-SPEI03}$ and $R_{NDVI-scPDSI}$. (a)-(d) show the spatial distribution of partial correlation coefficient for precipitation (a), shortwave radiation (b), temperature (c), and atmospheric CO_2 (d) in explaining $R_{NDVI-SPEI03}$; (e)-(h) show the spatial distribution of partial correlation coefficient for precipitation (e), shortwave radiation (f), temperature (g), and atmospheric CO_2 (h) in explaining $R_{NDVI-scPDSI}$.

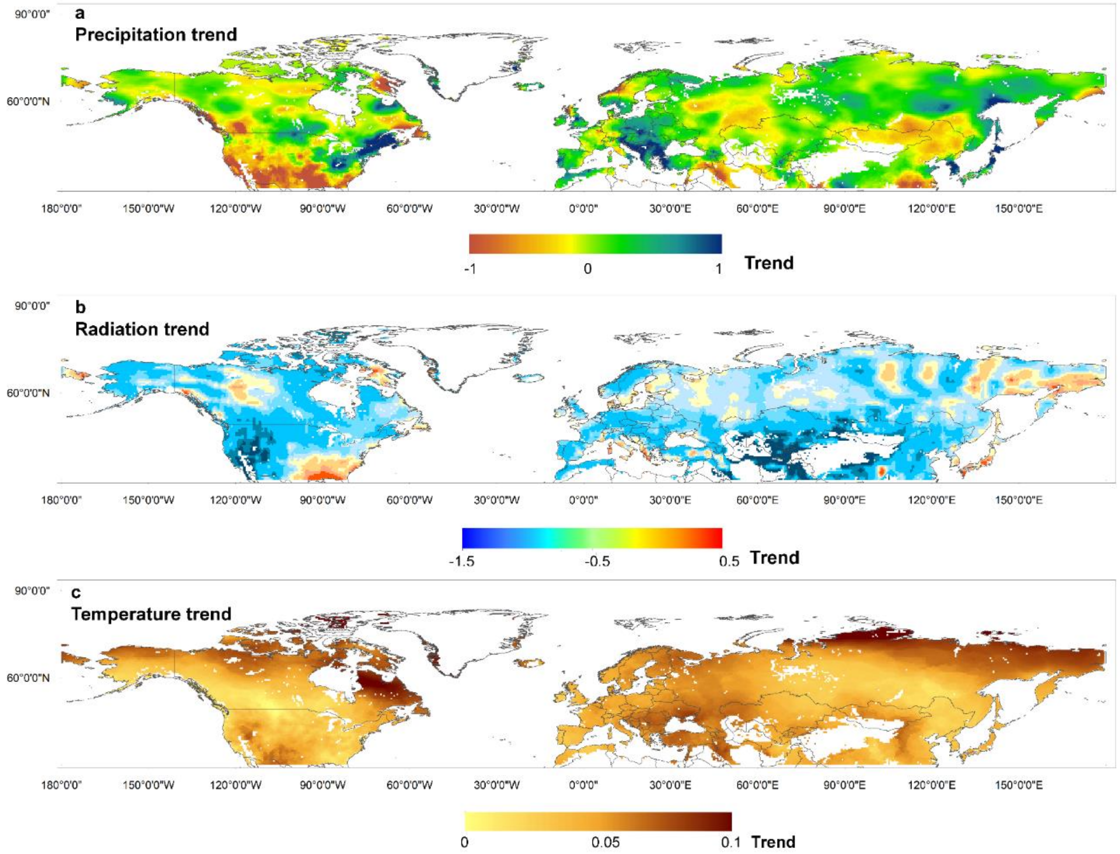

Supplementary Figure 13| Spatial distributions of interannual trends for precipitation (a), shortwave radiation (b), and temperature (c).

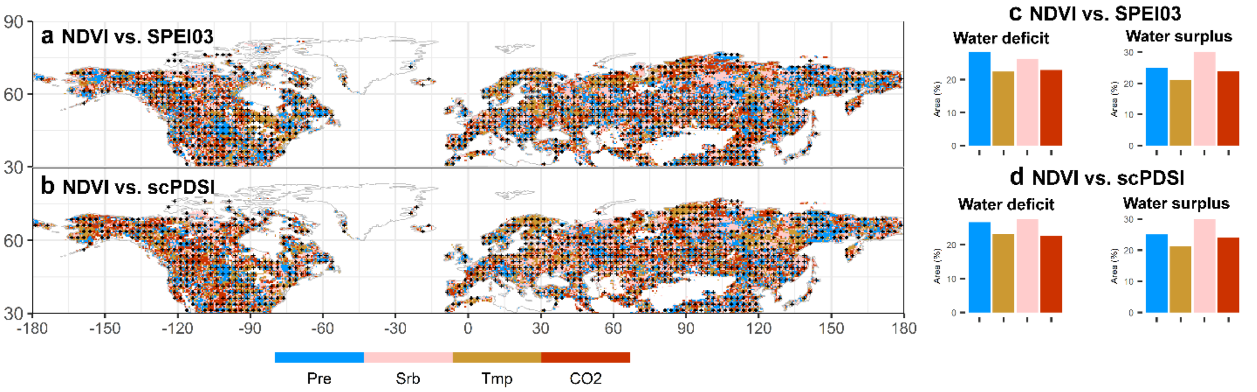

Supplementary Figure 14| Attribution of meteorological factors and atmospheric CO_2 to the correlations between normalized difference vegetation index (NDVI) anomaly and water availability indices over the last three decades based on 'relaimpo' relative importance algorithm. (a) and (b) are the spatial distributions of the dominant factor influencing $R_{NDVI\text{-}SPEI03}$ and $R_{NDVI\text{-}scPDSI}$, respectively. The dots show the regions that $R_{NDVI\text{-}SPEI03}$ or $R_{NDVI\text{-}scPDSI}$ variations are significantly explained by precipitation, radiation, temperature and atmospheric CO_2. (c) and (d) the percentage areas where the water deficit and water surplus responses can be explained by one of the four dominant factors (i.e., precipitation, temperature, radiation, and CO_2). Pre: precipitation; Rad: radiation; Tmp: temperature; CO_2: atmospheric CO_2.
